# Dataset of PY53 Nickel-Titanate yellow pigments for plastic, enamel and ceramic glaze applications

**DOI:** 10.1016/j.dib.2023.109040

**Published:** 2023-03-07

**Authors:** Emre Aslan, Emre Toy, Zeynep Güner, Emine Yeşilci, Alejandro Grijalbo, Buğra Çiçek

**Affiliations:** a Akcoat R&D Centre, Pigment Division, 2nd IZ, Sakarya 54300, Turkey; b Akcoat Advance Chemical Coating, El Colomer IP, Castellón 54300, Spain; c Yıldız Technical University, Department of Metallurgical and Material Science Engineering, İstanbul 34210, Turkey; d Istanbul Technical University, Chemical Engineering, İstanbul 34469, Turkey

**Keywords:** Nickel-Titanate, Color tone, Plastic application, Enamel application, Ceramic application, NiO

## Abstract

This dataset shows the change in color tone in plastic (masterbatch), enamel, and ceramic (glaze) colored with PY53 Nickel-Titanate-Pigment calcined with different NiO ratios by solid-state reaction. The pigments were mixed with milled frits and applied to the metal and ceramic substance for enamel and ceramic glaze applications, respectively. For the plastic application, the pigments were mixed with melted polypropylene (PP) and formed into plastic plates. The CIELAB color space approach was used to assess L*, a*, b* values over applications created for plastic, ceramic, and enamel trials. These data can be used to evaluate the color of PY53 Nickel-Titanate pigments with different NiO ratios in the applications.

**Specifications Table**

**Table:** 

Subject	Materials Science; Surfaces, Coatings and Films
Specific subject area	Color differences of pigments produced by solid-state reaction method used in Pigments, Glaze, Enamel coatings
Type of data	Table Figure
How the data were acquired	To evaluate the color values of the test samples, the Konica Minolta CM-700d Spectrophotometer was used employed. The Specular reflection excluded (SCE 10°-D65) approach was adopted since the material under test is more susceptible to surface conditions.
Data format	Raw
Description of data collection	UV-VIS spectrophotometer was used to measure the L*, a*, and b* color parameters to determine the impact of various NiO ratio on the color characteristics of the material. The CIE (Commission Internationale de l'Eclairage)- L*, a*, b* colorimetric technique was applied. L* represents the color's level of brightness and darkness on a scale ranging from white (L* =100) to black (L* =0). The scales a* and b* extend from the green (-a*) to the red (+a*) axis and the blue (-b*) to the yellow (+b*) axis, respectively.
Data source location	Data presented in this article is collected at Akcoat R&D Centre, Pigment Division, 2nd IZ, 54300, Sakarya, Turkey
Data accessibility	Repository name: Mendeley Data Data identification number: 10.17632/mmt5h4g23m.3 Direct URL to data: https://data.mendeley.com/datasets/mmt5h4g23m
Related research article	E. Aslan, E. Toy, Z. Güner, A. Grijalbo, E. Yeşilci, B. Çiçek, The effect of various nickel oxide ratios on the color and reflectivity of PY53 nickel-titanate yellow pigment, *Results in Chemistry,Volume 4, January 2022, 100662* (2022), https://doi.org/10.1016/j.rechem.2022.100662

## Value of the Data

• This [dataset] [4] is an important resource for those who want to see where the CIELAB coordinates are headed, showing the NiO ratio and post-application color tone values ​​for enamel, ceramic, ink and plastic (masterbatch) applications that use or develop PY53 inorganic pigment.
• This data can be used for people who develop, use, and apply pigment to get information about the color tone before application.
• This data can be used by researchers studying the ratio of metal oxides and the properties of the pigment formed by the solid-state reaction.
• This data can be used to understand the effect of metal oxide on the reflection properties of PY53 pigment for people studying %R reflection.
• This data can be used in future studies to investigate the effect of the studies to be made by changing the NiO ratios on the color tone and the effect in different applications.
• This data can be used as a source to find the starting formula for researchers who want to develop PY53 pigment*.*

## 1 Objective

Different nickel oxide ratios studies of inorganic PY53 Nickel-Titanate pigments are not available in the literature. All the work done is on a single application or on the synthesis of a single pigment. This [dataset] [4] allows the color tones of different applications of the pigment with different NiO ratio to be negotiated as described in the (Aslan et al.) [1].

## 2 Data Description

The [dataset] [4] is provided as an excel file (.xlsx) including 3 sheets.

‘’Plastic Application’’ sheet gives the results of the PY 53 pigment mixed with the polymer. ‘’Enamel Application’’ and ‘’Ceramic Glaze Application’’ sheets give the results of PY53 pigment of metal plates prepared with enamel frit.

**Fig. 1:**
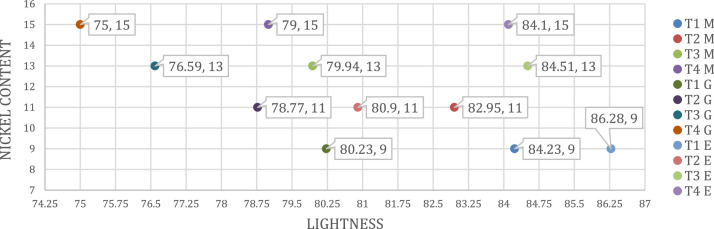
All applications CIELAB coordinates and comparison. M; Plastic (Masterbatch), G; Ceramic (Glaze), E; Enamel Applications (L* LIGHTNESS).

**Fig. 2:**
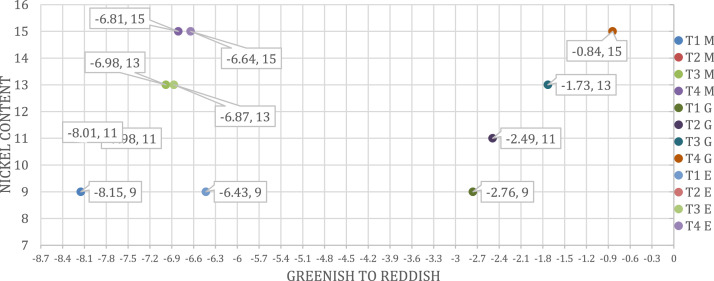
All applications CIELAB coordinates and comparison. M; Plastic (Masterbatch), G; Ceramic (Glaze), E; Enamel Applications (a* GREENISH TO REDDISH).

**Fig. 3:**
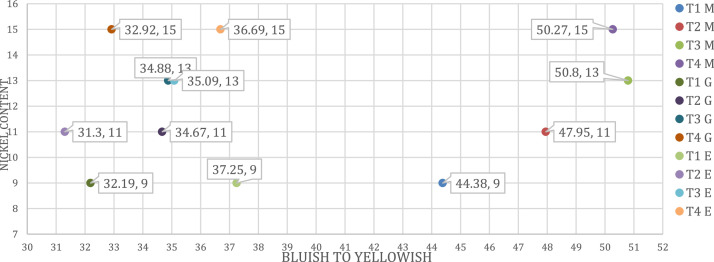
All applications CIELAB coordinates and comparison. M; Plastic (Masterbatch), G; Ceramic (Glaze), E; Enamel Applications (b* BLUISH TO YELLOWISH).

Table 1 shows the %wt. of Sb_2_O_3_/TiO_2_/NiO added together with the %wt. of excess NiO at different ratios. Tables 2, 3, 4 show the L*a*b* positions of different NiO containing samples in CEILAB color space for plastic (masterbatch), enamel and ceramic glaze applications. Figs. 1-2-3 shows their position in the color space of L* LIGHTNESS, a* (GREENISH TO REDDISH), b*(BLUISH TO YELLOWISH) which is a comparison of CIELAB coordinates for all applications.

**Table 1:** Formulation of Pigments %wt.

Sample	Sb_2_O_3_/TiO_2_/NiO (%wt)	Excess NiO (%wt)
T1	16/75/9	0
T2	16/75/9	2
T3	16/75/9	4
T4	16/75/9	6

**Table 2:** CIE L* a* b* values of the plastic(masterbatch) application.

L* a* b* D65 10^0^			
Sample	L*	a*	b*
T1	92.12 ± 0.01	-5.44 ± 0.01	20.07 ± 0.01
T2	91.53 ± 0.01	-5.03 ± 0.01	19.84 ± 0.01
T3	91.54 ± 0.01	-4.62 ± 0.01	19.5 ± 0.01
T4	92.7 ± 0.01	-4.62 ± 0.01	17.92 ± 0.01

**Table 3:** CIE L* a* b* values of the enamel application.

L* a* b* D65 10^0^			
Sample	L*	a*	b*
T1	86.28 ± 0.01	-6.43 ± 0.01	37.25 ± 0.01
T2	80.90 ± 0.01	-8.01 ± 0.01	31.30 ± 0.01
T3	84.51 ± 0.01	-6.87± 0.01	35.09 ± 0.01
T4	84.10 ± 0.01	-6.64 ± 0.01	36.69 ± 0.01

**Table 4:** CIE L* a* b* values of the ceramic glaze application.

L* a* b*	D65 10^0^		
Sample	L*	a*	b*
T1	80.23 ± 0.01	-2.76 ± 0.01	32.19 ± 0.01
T2	78.77 ± 0.01	-2.49 ± 0.01	34.67 ± 0.01
T3	76.59 ± 0.01	-1.73± 0.01	34.88 ± 0.01
T4	75 ± 0.01	-0.84 ± 0.01	32.92 ± 0.01

## 3 Experimental Design, Materials and Methods

### 3.1 Calcination process

The formulations of the samples were made with various NiO ratios are displayed in Table 1. TiO_2_ and Sb_2_O_3_ were maintained at constant values, whereas NiO was calcined at increasing ratios. The calcination was carried out in electric furnaces at a heating rate of 7°C/min at 1200°C for 1 h. The raw materials were subjected to a mixing process beforehand [3]. The particle sizes of pigments were reduced by grinding them in a Rosetti mill for 10 minutes [1].

### 3.2 Plastic (Masterbatch) application

After polypropylene (PP) was melted in the hot mixer at 180°C, the pigment was added into melted polymer. And then, press was applied on the melted polymer to obtain plastic plate to regulate the color tone in the plastic application [1], [2].

### 3.3 Enamel application

Glass-ceramic frit, water, kaolin, and pigment were all milled for 8 minutes in a Rosetti mill to observe the color values of the pigments with various NiO ratios in enamel application. With a spray gun the resultant aqueous combination was sprayed to the sheet metal. After the created plate was fired at 800°C, the color values were measured.

### 3.4 Ceramic glaze application

Ceramic frit, water, kaolin, and viscosity agents were added and processed with a Rosetti mill for 30 minutes to examine the color changes of various NiO ratios in ceramic glaze application. After preparing the glaze with 4% pigment added, it was applied on the ceramic tile with a slide. The thickness of glaze layer is 0.7 mm. The ceramic tile was then fired at 1100°C in a roller furnace.

### 3.5 CIELAB coordinates

The CIELAB coordinates for all applications are given in Figs. 1-Fig. 2, Fig. 3. The lightness changed as the nickel ratio changed. In plastic application, the density increased as the nickel ratio increased. In the enamel application, the color became redder as the nickel ratio increased. In the ceramic application, the color became more yellow as the nickel increased.

## Ethics Statements

This work does not involve any type of human studies, animal studies, or data gathered using social media.

## CRediT authorship contribution statement

**Emre Aslan:** Conceptualization, Writing – review & editing. **Emre Toy:** Methodology. **Zeynep Güner:** Validation. **Emine Yeşilci:** Data curation. **Alejandro Grijalbo:** Investigation, Resources. **Buğra Çiçek:** Project administration.

## Declaration of Competing Interest

The authors declare that they have no known competing financial interests or personal relationships that could have appeared to influence the work reported in this paper.

## Data Availability

Data from different applications of Nickel-Titanate yellow pigments (Original data) (Mendeley Data).
